# Efficacy of prolonged tapered and pulsed vancomycin regimen on recurrent *Clostridioides difficile* infection in the Japanese setting: a case control study

**DOI:** 10.1186/s40780-019-0147-1

**Published:** 2019-08-08

**Authors:** Takumi Umemura, Aiko Ota, Yoshikazu Mutoh, Chihiro Norizuki, Takahito Mizuno, Koji Kozaki, Yoshiaki Ikeda, Toshihiko Ichihara

**Affiliations:** 1 Department of Pharmacy, Tosei General Hospital, 160, Nishi oiwakecho, Seto, Aichi 489-8642 Japan; 2 Department of Infection and Prevention, Tosei General Hospital, 160, Nishi oiwakecho, Seto, Aichi 489-8642 Japan; 3 Department of Infectious Disease, Tosei General Hospital, 160, Nishi oiwakecho, Seto, Aichi 489-8642 Japan; 4 College of Pharmacy, Kinjo Gakuin University, 2-1723, Omori, Moriyama-ku, Nagoya, Aichi 463-8521 Japan

**Keywords:** Vancomycin, Vancomycin tapered and pulsed dose regimen, *Clostridioides difficile* infection

## Abstract

**Background:**

According to the Clinical Practice Guidelines for *Clostridioides difficile*, oral vancomycin is to be used in vancomycin tapered and pulsed regimen (VCM-TP) for recurrent *Clostridium difficile* infection (CDI). However, data on the efficacy of VCM-TP in Japanese patients with recurrent CDI are scarce. To address this gap, we investigated the efficacy of VCM-TP and performed a case-controlled study to assess the risk factors associated with treatment failure in these patients.

**Findings:**

We conducted this study on all patients who were administered VCM-TP for recurrent episodes of CDI between January 2008 and December 2018 at Tosei General Hospital. All patients had documented follow-ups within 90 days after completion of the VCM-TP. Data were obtained for comparative analysis of treatment success or failure. Thirty-six patients were eligible for this study, and treatment success was documented in 23 patients (63.9%) following VCM-TP treatment. Treatment success was documented in 22 of 30 (73.3%) patients who received the recommended therapy according to the Clinical Practice Guidelines. The frequency of patients treated with the recommended therapy was higher in the treatment success group (95.7%) than in the treatment failure group (61.5%) (OR: 13.75, 95% CI: 1.39–136.39, *p* = 0.016). Vancomycin-resistant enterococci culture tests were performed in 20 patients (55.6%), and all results were negative.

**Conclusions:**

Our findings suggest that VCM-TP is a good therapeutic option for recurrent CDI in Japanese patients. Furthermore, administration of the recommended VCM-TP is important for achieving a high rate of treatment success. Hence, antimicrobial stewardship teams should support the implementation of recommended VCM-TPs.

## Background

*Clostridioides difficile* infection (CDI) is one of the nosocomial infections associated with poor outcome [1, 2] and involves a huge medical expense [3, 4]. The 30-day mortality rate associated with CDI is 8%–31% [5], and the rate of recurrence is 16%–24% despite successful treatment of the initial episode [6]. After the first episode of recurrence, the rate of second CDI recurrence is 22.6%–41.8 [7], and the medical expenditure in patients with recurrent CDI is higher than that in patients with non-recurrent CDI [8]. According to the Clinical Practice Guidelines for *Clostridium difficile* by the Infectious Diseases Society of America (IDSA) and the Society for Healthcare Epidemiology of America (SHEA), oral vancomycin should be used in a vancomycin tapered and pulsed regimen (VCM-TP) for a standard 10-day course to treat the initial episode of CDI [9]. The Clinical Practice Guidelines by the Japanese Society of Chemotherapy and The Japanese Association for Infectious Diseases recommend using VCM-TP for the first or for ≥2 recurrence of CDI as an alternative therapy [10]. Despite reports of the efficacy of VCM-TP for recurrent CDI [11, 12], data on Japanese patients with recurrent CDI remain scarce. Therefore, we investigated the efficacy of VCM-TP in Japanese patients with recurrent CDI and performed a case-controlled study to assess the risk factors associated with treatment failure from recurrent CDI.

## Methods

### Study samples

This study was approved by the ethics committee of the Tosei General Hospital (receipt No. 769). This study was conducted on all patients who were administered VCM-TP for recurrent episodes of CDI (defined as symptoms of re-infection within 8 weeks of the prior episode) between January 2008 and December 2018 at Tosei General Hospital, a 633-bedded hospital. VCM-TP was defined as tapering vancomycin administration frequency in the sequence of once daily (pulse phase), and then every other day or every third day dosing for at least 2 weeks [13]. All patients had at least one of their prior CDI episodes confirmed by a positive test for the *C. difficile* toxin in their stool. For detecting the toxin, lateral flow immunoassay was performed in the stool specimens using Uniquick (Kanto Kagaku Kanto Kagaku Co., Ltd., Tokyo from January 2008 to June 2011), X/pect toxin A/B (Kanto Kagaku Co., Ltd., Tokyo from July 2011 to September 2014) and C. Diff Quik Chek Complete (Alere Medical Co. Ltd., Tokyo from October 2014 to December 2018).

### Efficacy of VCM-TP

All patients were followed up for more than 90 days after completion of VCM-TP. Treatment success was defined as the non-recurrence of diarrheal symptoms that require re-treatment with a CDI-specific agent [13]. Some patients noted a transient recurrence of symptoms near the end or just after completion of the pulse phase of the VCM-TP, and it resolved spontaneously, usually within a day; these episodes were not considered as recurrent CDI [13]. We investigated the treatment success rate in all patients with VCM-TP, in patients following the recommended VCM-TP according to the Clinical Practice Guidelines (125 mg four times per day for 10–14 days, two times per day for a week, once per day for a week, and every 2 or 3 days for 2–8 weeks) [9], and in patients following inappropriate VCM-TP therapy, which is defined as VCM-TP that departs from the recommended therapy.

### Factors associated with treatment failure in the administration of VCM-TP

The following data were obtained for comparative analysis of treatment success or failure: age, sex, underlying diseases (hypertension, diabetes mellitus, prior abdominal surgery, gastroesophageal reflux, immunocompromised, chronic kidney disease, chronic liver disease, and malignancy according to Sirbu et al. [13]), number of CDI episodes, duration of treatment, Charlson comorbidity index, drug use (proton pump inhibitors, histamine receptor-2 blockers, probiotics, antidiarrheals, and antibiotic use 90 days prior to the treatment), concomitant antibiotic use with VCM-TP treatment, disease severity (according to the Zar criteria and the MN criteria [14, 15]), and recommended VCM-TP according to the Clinical Practice Guidelines.

### Detection of vancomycin-resistant enterococci during or after VCM-TP

Stool culture tests for vancomycin-resistant enterococci (VRE) were performed between 14 days after initiation and 90 days after completion of VCM-TP to confirm the presence of VRE. The BD Vancomycin-Resistant Enterococci Selective Agar (Becton Dickinson©, NJ, USA) was used to detect VRE, and Microscan Walkaway system (Beckman Coulter, California, USA) was used to identify VRE at the species level and to determine antimicrobial susceptibility. All cultures were considered resistant according to the breakpoints defined by the Clinical & Laboratory Standards Institute (M100 S-22).

### Statistical analysis

The qualitative and stratified continuous variables were compared using Fisher Exact test or Pearson χ^2^ test. The continuous variables were compared using Mann–Whitney U test. The predictive values are presented as the odds ratios (ORs) with respective 95% confidence intervals (CI). Two-tailed *p* < 0.05 indicated statistical significance. All analyses were performed using IBM SPSS Statistics version 25 (IBM®).

## Results

### Efficacy of VCM-TP

**Fig. 1:**
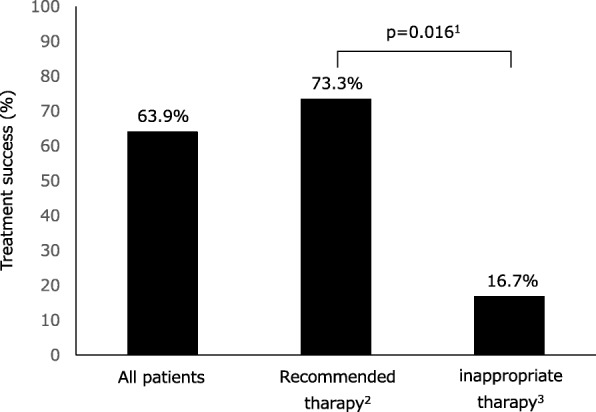
Efficacy of Vancomycin tapered and pulsed-dose regimen in patients with recurrent *Clostridioides difficile* infection. This shows the treatment success rate of all patients under the vancomycin tapered and pulsed-dose regimen (VCM-TP); patients with VCM-TP according to the IDSA guideline (recommended therapy); and patients with VCM-TP departing from the recommended therapy (inappropriate therapy). ^1^Fisher Exact test, ^2^vancomycin tapered and pulsed-dose regimen according to IDSA guideline, ^3^vancomycin tapered and pulsed-dose regimen not following the IDSA guideline

The study included 38 consecutive patients treated with VCM-TP for recurrent CDI. Two patients who died from unrelated disease before completing their regimens were excluded from the analysis. Table 1 shows the characteristics of the included patients. Among the remaining 36 patients, the median number of CDI episodes was 3 (range, 2–7). Twenty-nine patients were male, and the median age was 80.5 years. The median duration of treatment was 43 (IQR, 36.8–55) days. Treatment success occurred in 23 of 36 (63.9%) patients following VCM-TP and in 22 of 30 patients (73.3%) following the recommended therapy. On the contrary, in patients with inappropriate therapy, treatment success occurred in 1 of 6 patients (16.7%), which is less than the success rate of those following the recommended therapy (*p* = 0.016) (Fig. 1).

**Table 1:** Characteristics of patients with *Clostridioides difficile* infection treated with vancomycin tapered and pulsed-dose regimen

Items	*n* = 36
Male sex (%)	19 (52.8)
Age, median (IQR^1^)	80.5 (21–95)
No. of CDI episodes, mode (range)	3 (2–7)
Duration of treatment day, median (IQR^1^)	43 (36.8–55)
Underlying disease (%)	
Hypertension	12 (33.3)
Malignancy	10 (27.8)
Immunocompromised	9 (25.0)
Diabetes mellitus	5 (13.9)
Prior abdominal surgery	5 (13.9)
Chronic kidney disease	4 (11.1)
Chronic liver disease	1 (1.5)
Charlson comorbidity index, median (range)	2 (0–8)
Drug use (%)	
Proton pump inhibitors	16 (44.4)
Histamine receptor-2 blockers	3 (8.3)
Probiotics	31 (86.1)
Antidiarrheals	0 (0.0)
Antibiotic use prior 90 days	33 (91.7)
Disease severity (%)	
Zar criteria [14]	
> 2	5 (13.9)
≤ 2	31 (86.1)
MN criteria [15]	
mild	13 (36.1)
moderate	19 (52.8)
severe	3 (8.3)

^1^IQR (interquartile range)

### Factors associated with treatment failure after administration of VCM-TP

Table 2 shows the comparative characteristics of patients in the treatment success and failure groups. The proportion of patients treated with the recommended therapy was higher in the treatment success group than in the treatment failure group (OR: 13.75, 95% CI: 1.39–136.39, *p* = 0.016). No statistically significant difference was observed between the two groups. The reasons for inappropriate use of VCM-TP in treatment failure patients were the inability of the patients to take vancomycin two times a day for a week (3 cases) and unnecessary washout period during treatment (2 cases).

**Table 2:** Comparison of *Clostridioides difficile* infection patient characteristics in the treatment success and failure groups after the vancomycin tapered and pulsed-dose regimen

	Treatment success (*n* = 23)	Treatment failure (*n* = 13)	*p* value
Male sex (%)	12 (52.2)	7 (53.8)	0.549^2^
Age, median (IQR^1^)	80 (73–83.5)	83 (76–87)	0.281^3^
No. of CDI episodes, mode (range)	3 (2–7)	3 (2–7)	0.115^3^
Underlying disease			
Hypertension (%)	8 (34.8)	4 (30.8)	1.000^2^
Malignancy (%)	7 (30.4)	3 (23.1)	0.716^2^
Immunocompromised (%)	5 (21.7)	4 (30.8)	0.693^2^
Diabetes mellitus (%)	4 (17.4)	1 (7.7)	0.634^2^
Prior abdominal surgery (%)	3 (13.0)	2 (15.4)	1.000^2^
Chronic kidney disease (%)	3 (13.0)	1 (7.7)	1.000^2^
Chronic liver disease (%)	1 (4.3)	0 (0)	1.000^2^
Charlson comorbidity index, median (range)	2 (0–8)	2 (0–5)	–
Drug use			
Proton pump inhibitors (%)	10 (43.5)	6 (46.2)	0.877^4^
Histamine receptor-2 blockers (%)	3 (8.3)	0 (0.0)	0.288^2^
Probiotics (%)	19 (82.6)	12 (92.3)	0.634^2^
Antidiarrheals (%)	0 (0.0)	0 (0.0)	–
Antibiotic use prior 90 days (%)	21 (91.3)	12 (92.3)	1.000^2^
Penicillins	7	1	
Cephalosporins	6	4	
Carbapenems	6	3	
Quinolones	5	3	
Antitubercular	6	5	
Others	4	3	
Concomitant antibiotics during VCM-TP^5^ (%)	16 (69.6)	6 (46.2)	0.549^2^
Disease severity (%)			
Zar criteria [14]			0.166^4^
> 2	2 (8.7)	3 (23.1)	
≤ 2	21 (91.3)	10 (76.9)	
MN criteria [15]			0.373^4^
mild	6 (16.7)	7 (53.8)	
moderate	14 (60.9)	5 (38.5)	
severe	2 (8.7)	1 (7.7)	
Recommended VCM-TP^6^ (%)	22 (95.7)	8 (61.5)	0.016^2^

^1^IQR (interquartile range), ^2^Fisher Exact test, ^3^Mann-Whitney U test, ^4^Pearson χ2 test, ^5^VCM-TP (vancomycin tapered and pulsed-dose regimen), ^6^vancomycin tapered and pulsed-dose regimen according to IDSA guideline

### Detection of VRE during or after VCM-TP

VRE culture was performed in 20 patients (55.6%) after 41.9 ± 17.7 (mean ± standard deviation) days of VCM-TP treatment initiation, and all were negative.

## Discussion

VCM-TP is recommended for the treatment of recurrent CDI, but a few studies have evaluated the efficacy of VCM-TP in Japanese patients with recurrent CDI. Therefore, we investigated the efficacy of VCM-TP in Japanese patients with recurrent CDI and performed a case-controlled study to assess the risk factors associated with treatment failure from recurrent CDI.

VCM-TP for recurrent CDI resulted in clinical improvement in 63.9% of all patients, and 73.3% of patients under the recommended therapy had clinical improvements (Fig. 1). McFarland et al. and Sirbu et al. reported the cure rate of recurrent CDI with VCM-TP as 69.0% (20/29 cases) and 74.0% (74/100 cases), respectively [11, 13]. Therefore, the effects of the recommended VCM-TP therapy in Japanese patients are similar to its effects in non-Japanese patients. Furthermore, our results showed that inappropriate VCM-TP therapy, following unconventional guidelines, can increase the treatment failure rate (Fig. 1, Table 2). Mcfarland et al. hypothesized that the recommended VCM-TP allows gradual weeding out of *C. difficile* spores from the intestinal reservoir, resulting in low recurrence rate when pulsed doses of vancomycin are administered over an extended period (usually 3 weeks). Hence, we considered that inappropriate VCM-TP is more likely to result in treatment failure than recommended VCM-TP because *C. difficile* spores are not completely removed due to interruptions in treatment.

Repetitive cycles of antibiotic-free periods and antibiotic pulses may be an effective strategy for treating recurrent CDI [11]. We consider that inappropriate therapy that violates the optimal schedule can lead to unnecessary VCM washout periods, thereby increasing the risk of regrowth and relapse of CDI. Accordingly, antimicrobial stewardship teams should implement the recommended VCM-TP.

Tomas et al. reported that alterations to the indigenous microbiota responsible for colonization resistance to *C. difficile* and VRE persist during and after completion of tapering courses of vancomycin [16]. However, in our study, VRE was not detected in 20 patients in whom the VRE culture test was performed 41.9 days after treatment initiation. As our clinical data were limited, further studies in this regard are warranted.

There are certain limitations to this study. First, although our study was retrospective and observational, it only involved single-institutional data, and the sample size was inadequate. Additional prospective studies or clinical trials in multiple centers with larger numbers of patients are warranted. Second, it was difficult to confirm whether recurrent CDI was caused by the same *C. difficile* strain. The recurrence rate of CDI by the same bacterial strain is 45.8%–83.0% [17, 18]. Furthermore, if recurrent CDI was caused by the same bacterial strain, determining the specific cause is difficult considering the environmental factors or colonization in the intestinal tract.

## Conclusions

Our findings suggest that VCM-TP can be a good therapeutic option for recurrent CDI in Japanese settings. Administration of the recommended VCM-TP is important to ensure a high rate of treatment success. Hence, antimicrobial stewardship teams should support the implementation of recommended VCM-TPs.

## Data Availability

All data generated or analyzed in this study are included in this published article.

## Abbreviations

CDI: *Clostridioides (Clostridium) difficile* infection
CI: Confidence interval
IQR: Interquartile range.
OR: Odds ratio
VCM-TP: Vancomycin tapered and pulsed-dose regimen
VRE: Vancomycin-resistant enterococci
